# Supplementary material for: EnzML: multi-label prediction of enzyme classes using InterPro signatures
Source: BMC Bioinformatics. 2012 Apr 25;13:61. doi: 10.1186/1471-2105-13-61 (PMC3483700; doi:10.1186/1471-2105-13-61)
Supplement: Addtional file 5 — The Java code to format the data files, evaluate and predict. The file enzml_java_code.tar.gz contains the Java code used to format database data to ARFF and XML formats, to execute cross and train-test (jackknife) evaluations and to record evaluation results to database. More information is included in the readme.txt file and the Javadoc files. The code can be used with a MySQL database. To use a different database software, other JDBC drivers might be required. [file 1471-2105-13-61-S5.gz › java_code/enzml2011/doc/uk/ac/ed/inf/csb/taweka/test/mulan/MulanDataSetWriterTest.html]

MulanDataSetWriterTest


---


|  |  |  |
| --- | --- | --- |
| **Overview** \| **Package** \| **Class** \| **Use** \| **Tree** \| **Deprecated** \| **Index** \| **Help** | |  |
| **PREV CLASS**   **NEXT CLASS** | **FRAMES**    **NO FRAMES**     **All Classes** |  |
| SUMMARY: NESTED \| FIELD \| CONSTR \| METHOD | DETAIL: FIELD \| CONSTR \| METHOD |  |


---


## uk.ac.ed.inf.csb.taweka.test.mulan Class MulanDataSetWriterTest

```
java.lang.Object
  junit.framework.Assert
      junit.framework.TestCase
          uk.ac.ed.inf.csb.taweka.test.mulan.MulanDataSetWriterTest
```

**All Implemented Interfaces:**: junit.framework.Test

---

``` public class MulanDataSetWriterTest extends junit.framework.TestCase ```

Class

**Version:**
:   7 May 2010

**Author:**
:   Luna De Ferrari luna.deferrari-at-ed.ac.uk

---

| **Field Summary** | |
| --- | --- |
| `static java.lang.String` | `arffInstance1` |
| `static java.lang.String` | `arffInstance3` |
| `static java.lang.String` | `arffInstance4` |
| `static java.lang.String` | `ATT1_TO_STRING` |
| `static java.lang.String` | `ATT3_TO_STRING` |


**Constructor Summary**

`MulanDataSetWriterTest()`


| **Method Summary** | |
| --- | --- |
| `static java.lang.String` | `arff()` |
| `static MulanDataSetWriter` | `dataSet()` |
| `static java.lang.String` | `getArffFilePath()` |
| `static MulanDataSetWriter` | `getDataSet(java.lang.String dataSetName, java.util.Vector<java.lang.String> instances, java.util.Vector<java.lang.String> attributes, java.util.Vector<java.lang.String> classValues)` |
| `void` | `testCreateAttributesList()`             public void testInitialise() { MulanDataSetFactory data = MulanDataSetFactoryTest.dataSet(); Enumeration instances = data.getDataSet().enumerateInstances(); Instance instA = (SparseInstance) instances.nextElement(); // instA.setClassValue(DataSetFactoryTest.CLASS1); // arffInstance1 = "{0 1,1 1,4 1, 5 1}"; // att 1 assertEquals("1", instA.stringValue(0)); // att 2 assertEquals("1", instA.stringValue(1)); // att 3 assertEquals("0", instA.stringValue(2)); // att 4 assertEquals("0", instA.stringValue(3)); // class 1 assertEquals("1", instA.stringValue(4)); // class 2 assertEquals("1", instA.stringValue(5)); // class 3 assertEquals("0", instA.stringValue(6)); // arffInstance3 = "{2 1,6 1}"; Instance instb = (SparseInstance) instances.nextElement(); // att 1 assertEquals("0", instb.stringValue(0)); // att 2 assertEquals("0", instb.stringValue(1)); // att 3 assertEquals("1", instb.stringValue(2)); // att 4 assertEquals("0", instb.stringValue(3)); // class 1 assertEquals("0", instb.stringValue(4)); // class 2 assertEquals("0", instb.stringValue(5)); // class 3 assertEquals("1", instb.stringValue(6)); // arffInstance4 = "{3 1,5 1}"; Instance instc = (SparseInstance) instances.nextElement(); // att 1 assertEquals("0", instc.stringValue(0)); // att 2 assertEquals("0", instc.stringValue(1)); // att 3 assertEquals("0", instc.stringValue(2)); // att 4 assertEquals("1", instc.stringValue(3)); // class 1 assertEquals("0", instc.stringValue(4)); // class 2 assertEquals("1", instc.stringValue(5)); // class 3 assertEquals("0", instc.stringValue(6)); } |
| `void` | `testGetArff()` |
| `void` | `testInitialise()` |

**Methods inherited from class junit.framework.TestCase**

`countTestCases, getName, run, run, runBare, setName, toString`

**Methods inherited from class junit.framework.Assert**

`assertEquals, assertEquals, assertEquals, assertEquals, assertEquals, assertEquals, assertEquals, assertEquals, assertEquals, assertEquals, assertEquals, assertEquals, assertEquals, assertEquals, assertEquals, assertEquals, assertEquals, assertEquals, assertEquals, assertEquals, assertFalse, assertFalse, assertNotNull, assertNotNull, assertNotSame, assertNotSame, assertNull, assertNull, assertSame, assertSame, assertTrue, assertTrue, fail, fail, failNotEquals, failNotSame, failSame, format`

**Methods inherited from class java.lang.Object**

`equals, getClass, hashCode, notify, notifyAll, wait, wait, wait`

**Field Detail**

### arffInstance1

```
public static final java.lang.String arffInstance1
```

**See Also:**: Constant Field Values

---


### arffInstance3

```
public static final java.lang.String arffInstance3
```

**See Also:**: Constant Field Values

---


### arffInstance4

```
public static final java.lang.String arffInstance4
```

**See Also:**: Constant Field Values

---


### ATT1\_TO\_STRING

```
public static final java.lang.String ATT1_TO_STRING
```

**See Also:**: Constant Field Values

---


### ATT3\_TO\_STRING

```
public static final java.lang.String ATT3_TO_STRING
```

**See Also:**: Constant Field Values


**Constructor Detail**

### MulanDataSetWriterTest

```
public MulanDataSetWriterTest()
```


**Method Detail**

### testCreateAttributesList

```
public void testCreateAttributesList()
```

:   public void testInitialise() { MulanDataSetFactory data =
    MulanDataSetFactoryTest.dataSet(); Enumeration instances =
    data.getDataSet().enumerateInstances(); Instance instA = (SparseInstance)
    instances.nextElement(); //
    instA.setClassValue(DataSetFactoryTest.CLASS1);
    // arffInstance1 = "{0 1,1 1,4 1, 5 1}"; // att 1 assertEquals("1",
    instA.stringValue(0)); // att 2 assertEquals("1", instA.stringValue(1));
    // att 3 assertEquals("0", instA.stringValue(2)); // att 4
    assertEquals("0", instA.stringValue(3)); // class 1 assertEquals("1",
    instA.stringValue(4)); // class 2 assertEquals("1",
    instA.stringValue(5)); // class 3 assertEquals("0",
    instA.stringValue(6));
    // arffInstance3 = "{2 1,6 1}"; Instance instb = (SparseInstance)
    instances.nextElement(); // att 1 assertEquals("0",
    instb.stringValue(0)); // att 2 assertEquals("0", instb.stringValue(1));
    // att 3 assertEquals("1", instb.stringValue(2)); // att 4
    assertEquals("0", instb.stringValue(3)); // class 1 assertEquals("0",
    instb.stringValue(4)); // class 2 assertEquals("0",
    instb.stringValue(5)); // class 3 assertEquals("1",
    instb.stringValue(6));
    // arffInstance4 = "{3 1,5 1}"; Instance instc = (SparseInstance)
    instances.nextElement(); // att 1 assertEquals("0",
    instc.stringValue(0)); // att 2 assertEquals("0", instc.stringValue(1));
    // att 3 assertEquals("0", instc.stringValue(2)); // att 4
    assertEquals("1", instc.stringValue(3)); // class 1 assertEquals("0",
    instc.stringValue(4)); // class 2 assertEquals("1",
    instc.stringValue(5)); // class 3 assertEquals("0",
    instc.stringValue(6)); }

---


### testGetArff

```
public void testGetArff()
```

---


### testInitialise

```
public void testInitialise()
```

---


### arff

```
public static java.lang.String arff()
```

---


### dataSet

```
public static MulanDataSetWriter dataSet()
```

---


### getArffFilePath

```
public static java.lang.String getArffFilePath()
```

---


### getDataSet

```
public static MulanDataSetWriter getDataSet(java.lang.String dataSetName,
                                            java.util.Vector<java.lang.String> instances,
                                            java.util.Vector<java.lang.String> attributes,
                                            java.util.Vector<java.lang.String> classValues)
```


---


|  |  |  |
| --- | --- | --- |
| **Overview** \| **Package** \| **Class** \| **Use** \| **Tree** \| **Deprecated** \| **Index** \| **Help** | |  |
| **PREV CLASS**   **NEXT CLASS** | **FRAMES**    **NO FRAMES**     **All Classes** |  |
| SUMMARY: NESTED \| FIELD \| CONSTR \| METHOD | DETAIL: FIELD \| CONSTR \| METHOD |  |


---
